# On Abscess of the Liver

**Published:** 1829-04-01

**Authors:** 


XXVII.

HEPATIC ABSCESS.

It seldom happens that inflammation of the liver runs into suppuration in this country, where depletion is so speedily and actively employed in all inflammatory affections. Mr. Waldron, of Bath, has recently published an instance of this kind, in the second number of our Midland cotemporary.

The patient was a young lady, 21 years of age, who evinced symptoms of hepatic inflammation on the 21st of July, 1826, and was four times bled, besides leeches, purgation, blisters, mercury, and the other items of the medicamenta antiphlogistica. Nothing, however, would stop the inflammation, and by the 15th of August, a tumour with fluctuation was evident on the left lobe of the liver, attended with great inconvenience to the respiratory functions. A puncture was made, and large quantities of pus and bile flowed out. The relief was great—the discharge gradually subsided—and her health so far amended that, by December, she ventured out in the carriage. The consequences (as was supposed) of this exercise were fatal. "All her urgent symptoms returned with increased violence, and defied all attempts even at mitigation, and, on the 3d of March, 1827, she died."

On dissection, marks of the original abscess and puncture were discernible; but the liver was greatly enlarged, and was, in fact, a mere shell, its whole interior being excavated into a mighty abscess, containing some pints of matter. Violent Summer heats and habitual constipation were considered to be the causes of this disease.

We are disposed to doubt that this was a second abscess formed in consequence of the carriage exercise during convalescence from the primary one. We believe that the great abscess, which ultimately excavated the whole organ, was going on at the time, or, at all events, took its rise from the period at which the first abscess ripened. The immense discharge of bile which continued to flow for some days after the puncture, surprised Sir George Gibbes, as well as Mr. Waldron. Circumstances have come to our knowledge, on several occasions, which induce us to think that physiologists have formed a very erroneous estimate of the quantity of bile which is habitually or occasionally secreted by the liver. We are quite satisfied that, under states of excitement, or after torpor and congestion, the biliary apparatus will throw out some pints of bile in the course of the 24 hours, though the usual daily secretion may not be more than six or eight ounces. The abscess which was opened by Mr. Waldron, in the first instance, must have communicated with some of the large biliary or hepatic ducts, otherwise the flow of bile could not have been so great by the external wound.

---

XXVIII.

